# Anocutaneous advancement flap provides a quicker cure than fissurectomy in surgical treatment for chronic anal fissure—a retrospective, observational study

**DOI:** 10.1007/s00423-021-02227-4

**Published:** 2021-06-22

**Authors:** Edgar Hancke, Katrin Suchan, Knut Voelke

**Affiliations:** 1 Sektion Proktologie, Klinik für Allgemein-, Viszeral-, Thorax- und Gefäßchirurgie, Ketteler Krankenhaus Offenbach, Lichtenplattenweg 85, D-63071 Offenbach am Main, Germany; 2 Centrum Coloproctologie, Klinik Maingau Vom Roten Kreuz, Eschenheimer Anlage 7, 60316 Frankfurt am Main, Germany

**Keywords:** Chronic anal fissure, Anocutaneous advancement flap, Wound healing, Recurrent anal fissure

## Abstract

**Purpose:**

Anocutaneous advancement flap is a surgical procedure for the treatment of chronic anal fissures. This study aimed to assess the results of anocutaneous advancement flap in a consecutive cohort of patients.

**Methods:**

This is a retrospective, observational study. From 2000 to 2011, 481 patients had been operated for a single chronic anal fissure at the Maingau Clinic of the German Red Cross in Frankfurt am Main. The intention was to excise the fissure by fissurectomy (FIS) and then to cover the wound primarily with an anocutaneous advancement flap (AAF). The primary outcomes were resolution of symptoms and healing rates 1 month postoperatively. Secondary outcomes included incidences of early and late complications, postoperative incontinence, and recurrent fissure.

**Results:**

Anocutaneous advancement flap was performed in 455 (94.6%). In 26 (5.4%) patients, AAF failed due to lacking skin and the wound left open after FIS. One month postoperatively, half of the patients with AAF were free of symptoms (53.2%) with complete wound healing (47.9%). The incidence of early complications within 1 month postoperatively was 0.9% after AAF. From 1 month to 5 years after operation anal abscesses and fistula occurred in 2.9%. Mild symptoms of anal incontinence were recorded in 0.2% and recurrent chronic anal fissure in 3.3% of patients. Subgroup analysis revealed improved wound healing 1 month postoperatively in patients with AAF compared to FIS.

**Conclusion:**

Anocutaneous advancement flap is a very safe sphincter-sparing surgical option for CAF, provides a quicker cure than fissurectomy, and may be considered a good first-line surgical treatment option for chronic anal fissures if medical treatment failed.

## Introduction

Anal fissures are defined to be chronic on the basis of time (persistence beyond 8 weeks or more) and presence of secondary morphology (fibrotic induration of the fissure edges, sentinel tag, and hypertrophied anal papilla). If conservative treatment in patients with chronic anal fissure (CAF) fails, surgical treatment is recommended [1, 2]. Lateral internal sphincterotomy (LIS) is the most common surgical procedure for surgery of CAF with numerous studies and excellent long-term cure rates [3]. However, the long-term risk of post-sphincterotomy anal incontinence is not to be neglected with rates of 9 to 14% in two recent meta-analysis studies [4, 5]. In order to preserve the intact sphincter, CAF may be treated alternatively by fissurectomy (FIS) without impairing anal continence[6], but complete healing of the secondary wound may often be delayed to 10 or 15 weeks [7, 8]. To shorten healing time, primary wound closure after fissurectomy may be performed with anocutaneous or mucocutaneous advancement flaps [9–22]. Flap procedures are primarily proposed for recurrent chronic anal fissure previously treated with LIS and in multiparous and postdelivery women with low anal resting pressure [23, 24]. We report our experience with anocutaneous advancement flap (AAF) as first-line surgical treatment for CAF irrespective of the patient’s gender, anal tone, and fissure location.

## Materials and methods

### Patients

A consecutive series of patients undergoing surgical treatment for chronic anal fissure unresponsive to conservative management were studied. Patients were treated from 2000 onwards after implementing AAF in our surgical department as the preferred operation for CAF. Those operated on up to 2011 were included in the study to allow a minimum period of 5 years of observation postoperatively. Results of a total of 1139 patients from 2000 to 2011 were published previously [25]. In order to rule out any influence on wound healing and postoperative complications from additional pathology, a second analysis of the data was performed in the present study: 380 patients (33.3%) with multiple fissures or with anal abscess, fistula, hemorrhoids, or others and 278 patients (24.4%) with no follow-up 1 month postoperatively were excluded from further analysis. All patients had persistent symptomatic CAF with post-defecatory pain and/or bleeding after being treated conservatively for at least 12 weeks. The failure of conservative treatment and the patient’s desire led to surgical treatment. The diagnosis of chronic anal fissure was based on the presence of typical clinical features, such as visible horizontal fibers of the anal sphincter at the base of the lesion or fibrosis with or without sentinel pile.

### Data and outcome measures

#### Study design

The preoperative, postoperative, and surgical data of the patients were reviewed retrospectively from a medical database. This included age, gender, symptoms, diagnosis, comorbidities, fissure location, additional pathology, surgical procedures for CAF, postoperative complications, residual symptoms and fissure healing after a follow-up of 1 month postoperatively, development of postoperative anal abscess or fistula, anal incontinence and recurrence of CAF within at least 5 years after operation. There was no structured follow-up.

Primary outcome measures were the incidences of

- Patients without symptoms (1) and
- Patients with their wound completely healed (2) 1 month postoperatively. Complete healing was diagnosed by complete epithelization on follow-up proctoscopy.

Secondary outcome measures were incidences of

- Early complications within 1 month postoperatively (3),
- Late complications (4) from 1 month to 5 years after operation,
- Symptoms of anal incontinence (5), and
- Recurrent fissure (6)—defined as residual or recurrent chronic anal fissure.

### Surgical procedure

**Fig. 1:**
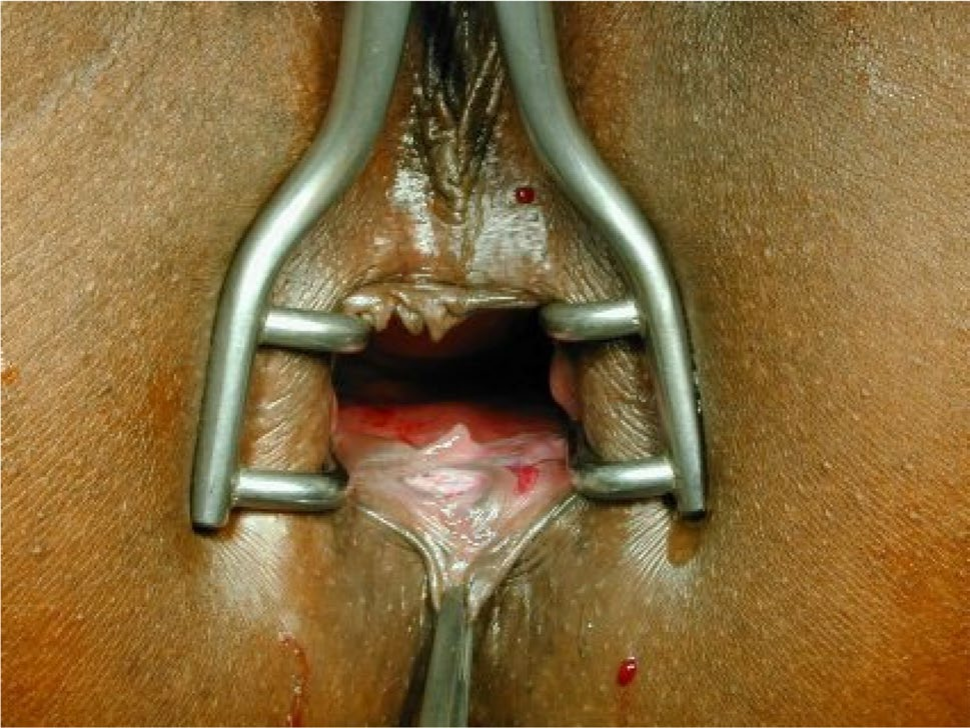
Chronic anal fissure at six o´clock supine position

**Fig. 2:**
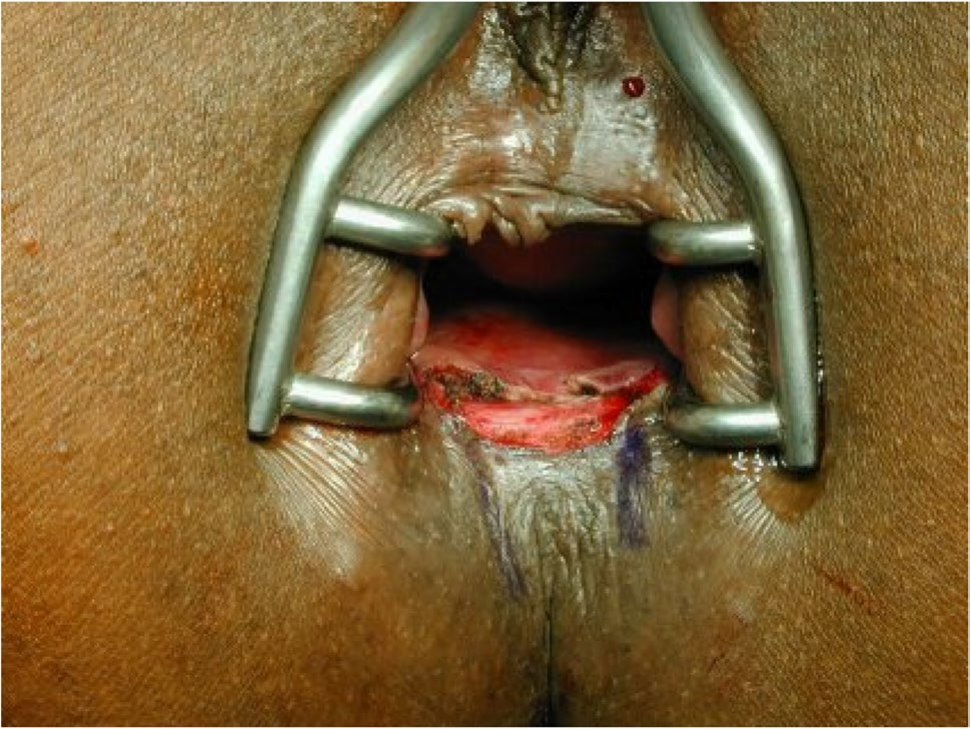
Fissurectomy

**Fig. 3:**
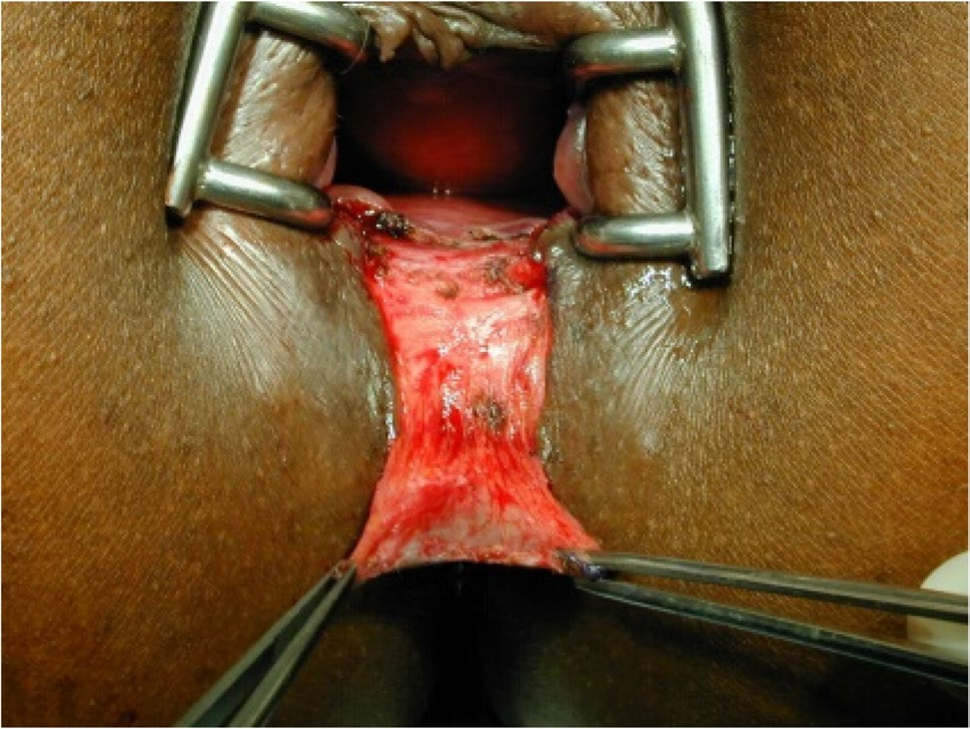
Rectangular flap prepared

**Fig. 4:**
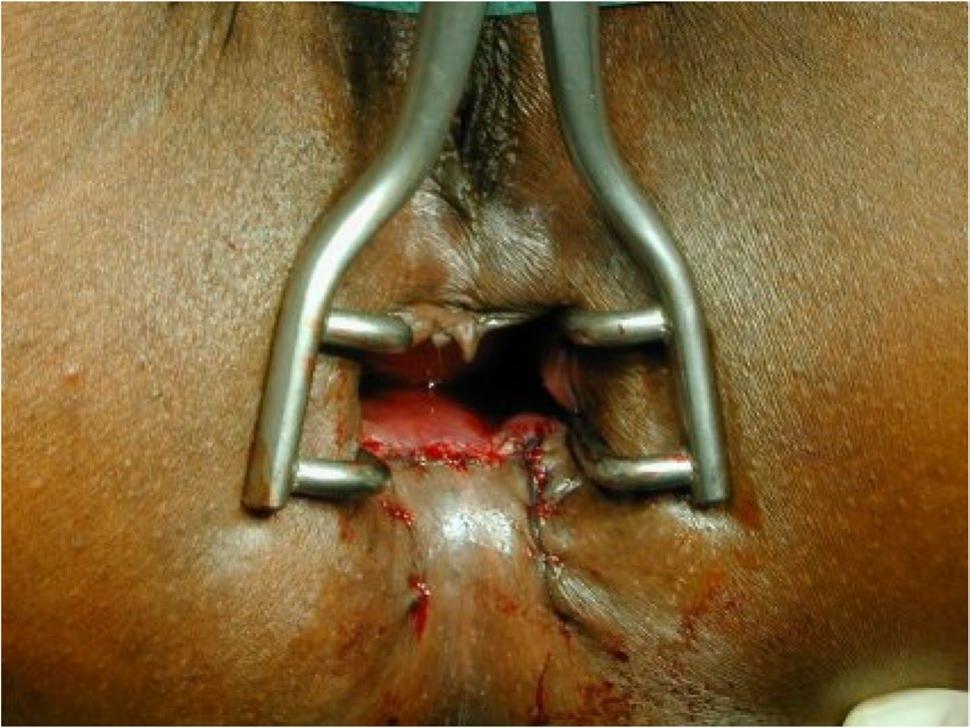
Flap advanced into the anal canal

The operations were performed by three proctologic surgeons (EH, KS, KV) under general anesthesia in a lithotomy position. Preoperatively, the rectum was cleaned by an enema. A single-shot antibiotic (cefazolin 2 g) was given intravenously at the time of skin incision. Local anesthesia of the perianal skin and a pudendal nerve block on both sides were performed with a total of 40 to 60 ml Naropin® (ropivacaine) or Carbostetin® (bupivacaine) before skin incision. A Sims (Schulze-Bergmann) rectal speculum was inserted in the anal canal. As described previously, fissurectomy was performed on all patients consisting of excision of CAF without diathermy. Any additional secondary changes such as skin tags, anal fibroma, and hyperplastic hemorrhoidal tissue were excised avoiding damage to the underlying anal sphincter [12]. Sphincterotomy was not performed. The wound was then covered with a u-shaped anocutaneous advancement flap. This included two parallel longitudinal incisions of the skin and preparing a rectangular skin flap which was raised and separated from the subcutaneous tissue (Figs. 1, 2, 3, and 4). The size of the flap was based on the width of the excision. The length–width ratio was not more than 1.5:1 in order to ensure an optimal vascular supply. The skin flap was transferred tension-free into the anal canal and sutured to the rectal mucosa with two continuous monofil sutures of 3–0 Monocryl® (Ethicon). No wound closure with a flap was performed after fissurectomy (FIS) in patients when a tension-free flap could not be raised due to lack of sufficient skin material.

### Postoperative management

Postoperative pain management included chamomile sitz baths, oral nonsteroidal anti-inflammatory drugs (Dexketoprofen 3 × 1 tablet per day), and additional pain medication, if required, with oral or intravenous dipyrone/paracetamol or tramadol drops/solution. The mean hospital stay was 2 days. Patients were advised to drink at least 2 l of water daily, eat fiber-containing food, and additionally take psyllium husks to soften stool. Patients with AAF and with FIS were advised to present postoperatively for a routine follow-up 1 month after surgery and later in any case of postoperative complication or recurrence.

### Statistics

The patient demographics, surgical, and pre- and postoperative follow-up data were extracted from the electronic patient documentation transferred to an Excel spreadsheet. The results were documented for all patients. A group of 26 patients were selected from 455 patients with AAF for subgroup analysis and compared to 26 patients with open wound healing because AAF failed after FIS. Both cohorts were identical in sample size, sex, age, comorbidities, previous operations, and fissure localization. A Chi Square Calculator for 2 × 2 double-sided test was used to assess differences between variables (https://www.socscistatistics.com/tests/chisquare/).

## Results

### Patient characteristics and clinical appearance of anal fissure

A total of 481 consecutive patients were surgically treated over a 12-year period for single CAF (Table 1). The majority of chronic anal fissures were located at the six o’clock lithotomy position.

**Table 1:** Patients

	N	%
Total	481	
Age (years) median; range	42.6; 19.8–87.0	
Sex		
Male	223	46.4
Female	258	53.6
Comorbidities		
Hypertension	73	15.2
Diabetes mellitus	25	5.2
Cardiovascular disease	18	3.7
HIV	12	2.5
Other (lung disease, liver cirrhosis, polyarthritis, sleep apnea)	7	1.5
Previous operations		
Chronic anal fissure	21	4.4
Anal fistula, hemorrhoids, marisques, thrombosis, condylomata	26	5.4
Localization of chronic anal fissure		
6 o’clock	370	76.9
12 o’clock	83	17.3
Other	28	5.8
Operation performed		
Anocutaneous advancement flap	455	94.6
Fissurectomy	26	5.4

### Surgery

Anocutaneous advancement flap was performed in 455 (94.6%) and fissurectomy in 26 (5.4%) of the patients.

### Symptom resolution and wound healing

One month postoperatively, 53.2% of the patients with AAF were free of symptoms with complete wound healing in 47.9% (Table 2).

**Table 2:** Postoperative outcome after anocutaneous advancement flap

	N	%
Total	455	
Results one month postoperatively		
No symptoms	242	53.2
Wound completely healed	218	47.9
Early complications (within 1 month)	4	0.9
Anal abscess	1	
Hemorrhoidal prolapse	1	
Wound dehiscence	1	
Urinary retention	1	
Late complications (from 1 month to 5 years)		
Anal abscess, fistula	13	2.9
Soiling	1	0.2
Recurrent chronic anal fissure	15	3.3

### Postoperative complications

Early complications within 1 month postoperatively were recorded in 0.9% of patients after AAF. Late complications from 1 month to 5 years postoperatively were anal abscesses and anal fistula in 2.9% of patients. One patient (0.2%) complained of mild symptoms of postoperative anal incontinence (soiling). Recurrent chronic anal fissure occurred in 3.3% of patients.

### Subgroup analysis comparing anocutaneous advancement flap to fissurectomy

In a subgroup analysis, 26 patients with AAF and 26 patients with FIS, corresponding in age, sex, comorbidities, and localization of anal fissure, were compared (Table 3). Duration of surgery was 26 min (16–40) in AAF and 15 min (7–35) in FIS. One month postoperatively, 12 patients with AAF (46.2%) and 2 patients with FIS (7.7%) showed complete wound healing (significant difference, chi-square test, p = 0.00177). There were no significant differences in the incidence of postoperative complications and recurrent fissures.

**Table 3:** Subgroup analysis—anocutaneous advancement flap (AAF) versus fissurectomy (FIS)

	AAF (n = 26)		FIS (n = 26)		P
	N	%	N	%	
Sex					
Male	17	65.4	17	65.4	
Female	9	34.6	9	34.6	
Age (years) median; range	48.6; 27.1–79.7		48.5; 24.5–81.2		
Comorbidities					
Hypertension	5		5		
Diabetes	2		2		
Cardiovascular disease	1		1		
HIV	4		4		
Other (sleep apnea)	0		1		
Previous operations					
CAF	1		2		
Hemorrhoids	2		2		
Localization of CAF					
6 o’clock	22		22		
12 o’clock	1		1		
Other	3		3		
Duration of surgery min (range)	26 (16–40)		15 (7–35)		
Results one month postoperatively					
No symptoms	15	57.7	8	30.8	0.0506
Symptoms	11	42.3	18	69.2	
Wound completely healed	12	46.2	2	7.7	0.00177*
Wound not healed	14	53.8	24	92.3	
Postoperative complications					
Anal abscess, fistula	2	7.7	3	11.5	0.6380
Recurrent chronic anal fissure	1	3.8	3	11.5	0.2979

*Chi-square statistic, significant at p < 0.01

## Discussion

Chronic anal fissures (CAF) nonresponsive to medical therapy may be treated with different surgical methods such as lateral internal sphincterotomy (LIS), anal stretch or balloon dilation (DIL), and fissurectomy (FIS) or with wound closure by advancement flaps (AAF) [26]. LIS is considered the treatment of choice for CAF with a strong recommendation based on high-quality evidence, 1A^1^, but can result in sphincter damage with fecal incontinence in 5 to 46% of patients postoperatively [2, 12, 27–29]. LIS may not be first-line therapy for patients such as women with prior obstetrical injuries and patients with irritable bowel syndrome, Crohn’s disease, previous anorectal operations, or a documented anal sphincter injury [2, 5, 23, 24]. The main alternative surgical method to LIS without damaging the anal sphincters is FIS. Randomized studies proved FIS to be equal in fissure healing and postoperative complications compared to LIS [30, 31]. Yet, complete wound healing is often delayed to several weeks after FIS [7, 8]. In order to shorten the time of wound healing after FIS, the wound may be covered by a flap. Primarily described to treat severe anal stenosis [32], anoplasty procedures have been performed to treat CAF. Techniques are mucosal advancement flaps from the rectum [16, 33, 34] and different techniques of anocutaneous advancement flaps from the perianal skin [9–15, 18, 19]. Most of the studies involving flaps have small patient numbers or excluded patients for different reasons [20, 35, 36]. In a large cohort of 1139 unselected patients operated for chronic anal fissures, AAF was performed in 80% of the patients and proved to be superior to FIS concerning postoperative symptoms and wound healing [25]. Yet, the study included patients with simultaneous pathologies like anal abscess, fistula, hemorrhoids, and others that needed additional surgery that may worsen the postoperative outcome. Therefore, in the present study, we focused only on patients treated surgically for single chronic anal fissures without any other pathology. AAF was performed in 95% of the patients; in 5%, the flap procedure could be not performed due to lack of sufficient skin material. Therefore, a limiting factor for creating a flap is too much tension on the mobilized flap in the presence of rigid skin. In a subgroup analysis, 46.2% of the patients with AAF showed complete wound healing 1 month after surgery compared to only 7.7% with FIS. This is the major advantage of AAF compared to FIS. In contrast, rectal mucosal flaps did not improve the time of wound healing compared to FIS [33]. Healing was achieved at a median of 7.5 weeks after rectal mucosal flap, meaning that half of the patients had their wound healed before 7.5 weeks and half of the patients later than 7.5 weeks*.* In our study, nearly half of the wounds had healed within 1 month (4.5 weeks) postoperatively which is 3 weeks earlier than following mucosal advancement flap. Early postoperative complications occurred in only 0.9% of our patients with AAF. Therefore, AAF can be considered a very safe surgical option for patients with CAF. Recurrences of CAF following AAF were 3.3% in patients observed up to at least 5 years postoperatively, comparable to results reported for LIS [4]. Only one patient (0.2%) complained of symptoms of postoperative anal incontinence (soiling). Therefore, AAF can be recommended as a sphincter-sparing surgical procedure for CAF if medical treatment fails. LIS may be used in patients with high sphincter tone or as a secondary surgical option for recurrent CAF.

Excessive bowel cleaning before AAF is not necessary, except for an enema 30 min before operation. We believe a single dose of intravenous antibiotics (cefazoline 1 g) during induction of general anesthesia is advised to prevent graft infection, although there is no study to support this yet. The main objection to flap procedures is the prolonged surgical time needed for the mandatory meticulous preparation of the flap. This may be an essential reason why AAF is not a frequently used technique and is reserved for patients at higher risk for postoperative incontinence disorders. In the present study, the surgical time was 26 min in AAF compared to 15 min in FIS. We regard the additional 10-min time being well worth to ensure quicker resolution of symptoms and wound healing after AAF. Most general surgeons performing proctologic interventions are familiar with the technique of advancement flaps in the surgical treatment of anorectal fistulas. Therefore, as AAF does not compromise the anal sphincter, it should be considered the first-line treatment option for patients with CAF despite the slight disadvantage of increased surgical duration.

### Limitations

The main limitations of this study are the retrospective design and the non-structured follow-up. However, we do not think that there is an influence on the results because both patients with AAF and with FIS were advised equally to present postoperatively in any case of complications or recurrences. Therefore, we believe that the results of our retrospective trial are valid without a bias. In order to confirm the results, a prospective randomized study comparing the outcome of AAF and FIS should be designed.

## Conclusions

AAF is a very safe sphincter-sparing surgical option for CAF and may be a good first-line treatment if medical treatment failed. The flap procedure requires experience in proctologic surgery and short additional surgical time. This seems justifiable since it provides a quicker wound healing than fissurectomy and is very rarely complicated by postoperative incontinence.

## Data Availability

All data are explicitly shown in tables.

## Declarations

### Permissions

Figures that have not been published elsewhere, no permission required.
